# Alzheimer’s Disease as a Major Public Health Concern: Role of Dietary Saponins in Mitigating Neurodegenerative Disorders and Their Underlying Mechanisms

**DOI:** 10.3390/molecules27206804

**Published:** 2022-10-11

**Authors:** Asaad A. Abduljawad, Mohammed Ahmed Elawad, Modawy Elnour Modawy Elkhalifa, Alshebli Ahmed, Alashary Adam Eisa Hamdoon, Liga Hasan Mohammed Salim, Muhammad Ashraf, Muhammad Ayaz, Syed Shams ul Hassan, Simona Bungau

**Affiliations:** 1 Public Health Department, Health Sciences College at Al Leith, Umm Al Qura University, Mecca 24382, Saudi Arabia; 2 Faculty of Public and Environmental Health, University of Khartoum, Khartoum 11115, Sudan; 3 Department of Pharmacy, Faculty of Biological Sciences, University of Malakand, Chakdara 18000, Pakistan; 4 Shanghai Key Laboratory for Molecular Engineering of Chiral Drugs, School of Pharmacy, Shanghai Jiao Tong University, Shanghai 200240, China; 5 Department of Natural Product Chemistry, School of Pharmacy, Shanghai Jiao Tong University, Shanghai 200240, China; 6 Department of Pharmacy, Faculty of Medicine and Pharmacy, University of Oradea, 410028 Oradea, Romania

**Keywords:** saponins, neurodegenerative disorders, Alzheimer’s disease, signaling pathways, neurotrophins

## Abstract

Saponins are triterpenoid or steroidal glycosides and are an important group of naturally occurring compounds of plant origin. They exhibit diverse pharmacological potentials including radical scavenging, as well as neuroprotective, anti-diabetic and anti-inflammatory activities, owing to their diverse chemical scaffolds. Saponins consist of an aglycone part (non-sugar) and a glycone part (sugar) and have at least one glycosidic (C–O sugar bond) linkage present between the glycone and aglycone mostly at C-3. On the basis of the aglycone part, saponins are classified into triterpenoid glycosides, steroid glycosides and alkaloid glycosides. Saponins exhibit neuroprotective activities against various disorders of the central nervous system (CNS) including stroke, Alzheimer’s disease (AD), Huntington’s disease (HD) and Parkinson’s disease (PD). They mediate their therapeutic effects by modulation of various pathological targets. This study highlights various neuroprotective mechanisms of saponins including free radical scavenging, modulation of neuroprotective signaling pathways, activation of neurotrophic factors, modulation of neurotransmitters, inhibition of BACE1 enzyme and tau hyper-phosphorylation. The study concludes that saponins have considerable efficacy against various pathological targets of neurological disorders, especially AD, and might be an important source of leads against neurodegenerative disorders.

## 1. Introduction

Alzheimer’s disease (AD) is a highly prevalent neurological disorder of old age and associated with behavioral and cognitive complications [1,2]. The symptoms of AD are not confined to the loss of memory and cognition, but it also associated with some non-cognitive characteristics such as depression, inability to perform routine daily activities and behavioral disturbances [3,4]. AD has a greater impact on the patients’ quality of life and function. It is observed that the rate of health service utilization among AD patients is very high and patients go through more falls and accidents as compared to age-matched controls [5,6]. With continuous decline in their independence, the person with AD puts large financial, psychological and physical burden on their family caregivers [7]. Subsequently, they are admitted more frequently into nursing homes, residential care and geropsychiatric hospitals. There are various estimates regarding the total cost of AD care. According to one mid-range estimate, the annual per-patient cost is $38,000, excluding the losses due to unemployment, premature deaths and morbidity [8]. With the passage of time, there is an expected increase in these costs proportionally with increase in the number of AD patients and severity of the disease [8]. 

## 2. Global Prevalence and Risk Factors of AD

Alzheimer’s disease (AD) is a highly prevalent neurodegenerative disorder of the old age and is the sixth leading cause of death in United States (US) [9,10]. According to Alzheimer’s Association report 2020, there are 5.8 million AD–dementia patients in US which is estimated to reach 13.8 million by 2050 [11]. The global prevalence of AD is estimated to be 35 million [12]. A study aimed to evaluate the actual and estimated prevalence of AD–dementia in US from 2010–2050 using longitudinal population-based analysis [13]. Results of the study revealed that there were 4.7 million people over 65 years of age who were suffering from AD–dementia in 2010. Among the sufferers, 0.7 million patients were of 65–74 years age, 2.3 million were 75–84 years and 1.8 million were 85 years or older. The projected number of AD patients is 13.8 million by the year 2050 [13]. 

Among the major risk factors for the development of AD is old age. Studies suggest that the disease prevalence is high among the older population of 65 years age and above [14,15]. With aging, the rate of incidence continues to increase and prevalence at the eighth decade of patients’ lives is where it peaks [16]. For instance, in persons of an age between 75 and 85 years, about 43% of them are at high risk for the development of AD. The prevalence of AD is found to be higher among women as compared to men [17]. Mostly, this difference is related to a lower rate of mortality at earlier ages in women as compared to men [16,18].

## 3. Pathological Targets of Alzheimer’s Disease

### 3.1. Brain Structure Alterations

On a macro level, the pathology of AD can be determined as the progressive loss of brain tissues. With the passage of time, the neuron loses its integrity and ultimately dies in a specific way with the disease progression. The first sign of AD is loss of memory, especially the short term memory. The part of the brain concerned with the memory is the cortex and hippocampus [19]. In the entorhinal cortex, which links the hippocampus (involved in the formation of memory) with the cerebral cortex, the AD symptoms start initially [19]. Magnetic resonance imaging (MRI) studies revealed that the loss of neurons begins before the loss of memory occurs. As atrophy occur in the brain tissues, the cerebrospinal fluid (CSF) fills the empty spaces of the brain tissues. The memory loss is more prominent in case of mild to moderate AD. The patient is unable to recall famous names as well as suffers confusion about well-known places. There is a change in the personality of the patient and their mood. Additionally, the patient is unable to perform complex work. After this, the atrophy expands to other areas in the cerebral cortex [19]. As the AD progresses, atrophy occurs in the area of the cortex, which controls sensory processing, reasoning, conscious thoughts and speech, and the symptoms of AD such as long term memory impairment, weight loss, seizures, unable to recognize loved ones, and incontinence become severe [19,20].

### 3.2. Progression of Degeneration in Alzheimer’s Disease

Among the hallmarks of the AD is the deposition of amyloid plaques (Aβ), highly phosphorylated tau proteins and intracellular deposition of neurofibrillary tangles (NFTs) [4,21]. During the course of AD progression, the amyloid plaques (Aβ) and NFTs are generated in the brain. These plaques and NFTs are deposited in various parts of the brain implicated in the cognition process and hamper the impulse transmission as well as initiate neurodegeneration. The Aβ plaques are considered to be responsible for the pathogenesis of AD (amyloid hypothesis) [22,23]. Aβ is known as mitochondrial poison, which, after fabrication, localizes to mitochondrial membranes and blocks the nuclear-encoded mitochondrial protein transport to mitochondria [24]. They also interact with various mitochondrial proteins, disrupt the electron transport chain and promote the excessive production of free radicals. Thus, these process cause mitochondrial damage, and initiate inflammatory processes in the neurons. Excessive amount of free radicals readily attack various biological molecules, including neurons, and also cause mutations [25]. 

#### 3.2.1. The β-Amyloid Hypothesis

**Figure 1:**
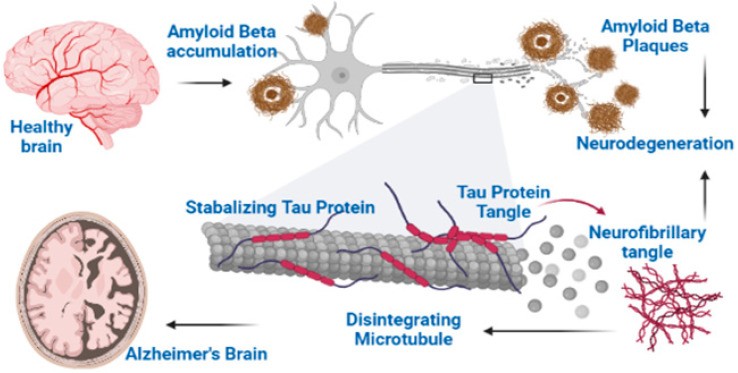
Two major hallmarks of Alzheimer’s disease: Aβ and NFTs.

The Aβ plaques are insoluble peptides which are formed from the abnormal segmentation of APP (amyloid precursor proteins) [26] (Figure 1). The actual contribution of these plaques in the development of the disease is not fully understood yet. Three enzymes including α-secretase, β-secretase and γ-secretase catalyze the metabolism of APP to give various products. Normally, the products of β-secretase (BACE1) are subsequently metabolized by γ-secretase, thus resulting in the formation of soluble peptides composed of 40 amino acids [27]. However, in AD, γ-secretase forms a variant which causes the cleavage of APP in abnormal way and produces an insoluble peptide of 42 amino acids which is called as Aβ_42_ or Aβ. The Aβ peptides clumps together to form aggregates called β-amyloid plaques. The α-secretase enzyme produces protective action because it causes the cleavage of APP at specific sites, which forbids the formation of Aβ [28].

#### 3.2.2. APP Mutations Promote Longer Aβ Formation

In the brain of AD patients, there is deposition of 39–43 amino acid peptides (Aβ_39–43_) in the form of Aβ plaques, which are derived from 677–770 amino acid proteins, collectively known as βAPP. Evidence indicates that amyloid has a crucial role in AD development. It arises from the recognition of FAD (Familial AD), in which the phenotypes of AD co-segregate with βAPP gene mutations. About three of the FAD-linked βAPP mutations transform the valine located three residues down from the carboxyl of Aβ_43_ (Val^717^ in βAPP_770_) to glycine (∆G), phenylalanine (∆F) or isoleucine (∆I). A fourth double mutation (∆NL) changes the lysine–methionine located immediately to Aβ_1_ (Lys^670^–Met^671^ in βAPP^770^) to asparagine–leucine. The positions of these mutations proposed that it may contribute to the development of AD by changing the processing of βAPP in such a manner which is amyloidogenic [29,30].

About five to six times more 4-kD Aβ is secreted by βAPP_∆NL_-expressing cells as compared to βAPP wild-type cells. Hence, the βAPP_∆NL_ experiences change in the processing which increases the deposition of amyloid. However, the transfected cells showing βAPP_695∆I_ do not release the excessive quantity of Aβ. From these observations it was found that FAD- linked mutations presenting on the carboxyl side of Aβ (∆F, ∆I, ∆G) change their cleavage to prefer longer Aβ generation, such as Aβ_1–42_ or Aβ_1–43._ As this longer β1–42 rapidly forms the amyloid fibrils as compared to Aβ_1–40,_ shifting of the cleavage site may cause the deposition of amyloid without enhancing the overall quantity of produced Aβ [29].

#### 3.2.3. Neurofibrillary Tangles (NFTs)

Neurofibrillary Tangles (NFTs) comprise abnormal bundles of filaments concentrated in neuronal axons, dendrites and perikarya [5]. Ultrastructure of these filaments demonstrates some regular constriction or having a straight appearance and these two filaments are twisted helicoidally around each other; this is why it is also named as PHF (paired helical filaments). From more studies it is found that PHF looks like a twisted ribbon, while its core cross section shows two C-shaped units. The thread of a neuropil consists of small-sized curly and dystrophic neurites that are scattered in neuropils and holding some abnormal filaments [31,32].

The silver staining methods are used for identification of NFT’s classically and can also be identified through green birefringence after staining with Congo Red, and also through S staining with thioflavin. For the detection of neurofibrillary lesions, the more reproducible method is immunocytochemical labeling with antibodies to tau proteins (the principle part of PHF). 

Few morphologically different types of NFTs can be differentiated, which mostly corresponds to different stages of evolution. The pre-tangle stage is characterized by phosphorylated tau accumulation in the compartment of somatodendrites, without PHF formation, while in dendrites and soma, some tau-immunoreactive rods appear at later stages. They correspond to the neuropil thread and NFTs, and are also detected through silver staining technique. The classical NFTs are formed from bundles that are packed tightly, which occupy a less or more important cell body part and extend into dendrites. The partial disaggregation of NFTs causes neuronal death. The extracellular tangle, which represents neuronal loss, remains apparently for a long period due to their partial resistance to proteolysis, despite of lack of an N-terminal domain in tau [32].

#### 3.2.4. The Cholinergic Hypothesis 

Another cause of AD pathogenesis is the cholinergic neuronal loss, which leads to a gradual decline in the number of cholinergic neurons [33]. Usually at later stages of AD, greater than 75% neuronal loss in some parts of the brain has been observed. Acetylcholine (ACh) acts as a neurotransmitter in the brain that is involved in impulse transmission and memory. The loss of cholinergic neurons is directly linked with cognition impairment. Acetylcholine (ACh) produces its effect by binding with two types of postsynaptic receptors, which are nicotinic and muscarinic receptors. The release of neurotransmitters from presynaptic neurons is caused by the binding of ACh to presynaptic nicotinic receptors. These neurotransmitters have a significant role in mood and memory, and include serotonin, acetylcholine, norepinephrine and glutamate; all of these are implicated in the pathology of AD [34].

#### 3.2.5. Oxidative Stress and Alzheimer’s Disease

In AD patients’ brains, the Aβ causes peroxidation of lipids and produces oxygen and nitrogen reactive species. To gain stability, these reactive species react with some other molecules. In this process, free radicals (high-energy electrons) are released and the reactive species make molecular bonds with the other molecules. As this is a permanent reaction, when the reactive species are attached to certain biological molecules, this alters them both structurally and functionally [35,36] and causes genetic mutations. The damage caused by oxidation occurs in every type of macromolecules including neurons (proteins, carbohydrates, lipids and nucleic acids). The brain is more sensitive to oxidative stress damage due to its rich lipid contents, high rate of oxygen consumption and low amount of antioxidant enzymes. This oxidation process in neurons causes various problems including non-recoverable DNA damage and upregulation of pro-inflammatory cytokines [37,38]. During the early stages of AD, the role of oxidative stress is very crucial, since it is linked temporally to the development of NFTs and amyloid plaques [39]. 

#### 3.2.6. Chronic Inflammation Hypothesis

Initially, the presence of immune system cells and antigens around the Aβ aggregates and tau proteins in AD patients brain signals the involvement of inflammatory process in the pathogenesis of AD [40]. The hypothesis is supported by epidemiological evidences, whereby it was observed that patients using anti-inflammatory agents for the management of chronic inflammatory diseases such as arthritis have a lower prevalence of AD [41]. However, it is unlikely that inflammation is the sole cause of AD, but it is definitely implicated in the progression of the disease [42]. For instance, the involvement of inflammatory mediators in the progression of AD is now well known [43]. In AD patients’ brains, the accumulation of Aβ causes the activation of microglia and initiates the pro-inflammatory cascades. Subsequently, potentially neurotoxic chemicals such as cytokines, chemokines, reactive nitrogen species (RNS), reactive oxygen species (ROS), as well as proteolytic enzymes, are liberated, which initiate neuronal degeneration [44,45]. Additionally, the activated microglia cause phosphorylation of tau proteins, which subsequently leads to the formation and neuronal accumulation of NFTs [46,47]. 

The inflammatory hypothesis in AD also suggests that polymorphisms in the genes regulating the inflammatory process cause sporadic AD [48]. For instance, the presence of polymorphic genes encoding IL-1 and TNFα were reported to cause excessive activation of microglia among AD patients [49,50]. Yet, the meta-analysis performed on genetic influences among AD patients has not supported the role of cytokine variations in the context of risk factors for AD. Though the role of APOE gene polymorphisms, especially in APOEe4 carriers, is suggested to be involved, its exact mechanism remains unclear [51,52]. 

#### 3.2.7. Other Neurotransmitters Deficiency

During AD, ACh, norepinephrine, and serotonin production is altered in the cerebral cortex of the brain. As serotonin is known to play a vital role in anxiety and depression, thus, depression is comorbid with AD. In AD brains, the quantity of serotonin receptors and transporters is changed, which corresponds to a gradual decline in cognitive performance and causes anxiety. The level of norepinephrine is also decreased and its neurons are lost in AD [53]. In addition to loss of memory, norepinephrine has been considered to be responsible for the psychological and behavioral symptoms of dementia (psychosis, agitation and aggression) [54].

## 4. Current Therapeutics against Alzheimer’s Disease

Unfortunately, drug discovery against AD is very slow and the clinically approved anti-AD therapeutic agents are limited to the use of AChE/BChE inhibitors only. Only four anti-AD agents are clinically approved, which include galanthamine (Reminyls^®^), donepezil (Aricept^®^), tacrine (Cognex^®^) and rivastigmine (Exelon^®^) [55,56]. The pharmacological action of these agents is mediated via boosting the ACh levels at the synapses of the brain via the inhibition of AChE/BChE enzymes. However, they do not eradicate the disease completely but slow down the disease progression and relieve disease symptomatology. 

Unfortunately, anti-AD drug discovery is very limited. According to the 2021 anti-AD drug discovery reports, about 126 agents were subjected to 152 clinical trials for AD. Among the tested compounds, 2 agents are in Phase III clinical trials, 74 agents are in Phase II clinical trials, whereas 24 compounds are in Phase I clinical trials. Among the tested agents, 82.5% are aimed to target the underlying biology of AD. Among the rest, 10.3% are aimed to augment cognitive performance, whereas 7.1% were tested for their efficacy in modulating the neuropsychiatric symptoms [9]. 

### Plant-Based Alzheimer’s Disease Therapeutics

Currently available AD agents are associated with severe side effects and have limited efficacy [5]. There is a need for an alternative therapy with no or fewer side effects, preferably from natural sources. The clinical approval of plant-derived compounds including galanthamine signifies the role of natural products in anti-AD drug discovery [57]. Numerous natural products and their derived compounds have been extensively studied for their efficacy to increase the memory of AD patients [58,59,60]. For instance, curcumin has shown pre-clinical efficacy on multiple targets of AD including inhibition of inflammatory pathways, Aβ deposition and improvements in cognitive performance [61,62,63]. Natural flavonoids such as catechins, myricetin and gossypetin inhibit Aβ aggregation, scavenge free radicals and inhibit vital enzymes implicated in AD [64,65,66]. Herbal medicines are considered to be beneficial to treat AD, with fewer side effects than currently available drugs. Of particular interest are ethnopharmacological-based compounds, with already-proven safety among local communities [67,68,69,70]. 

## 5. Saponins and Their Neuro-Pharmacological Properties

Saponins represent a group of naturally occurring organic compounds distributed in the kingdom plantae [71]. In the pharmaceutical industry, the saponins are used as precursors for the semi-synthesis of steroidal compounds. Saponin-rich plants are used for various purposes and are considered as the main ingredients in Traditional Chinese Medicine (TCM); these plants notably include ginseng [72]. Saponins exhibit diverse pharmacological properties including antioxidant, anti-neuroinflammatory and neuro-cognitive benefits [73,74,75]. Several studies reported the neuroprotective potentials of saponins. Recently, sarsasapogenin isolated from *Asparaguss racemosus* was reported to inhibit cholinesterases (AChE IC_50_ 9.9 µM, BChE IC_50_ 5.4 µM), beta amyloid-cleaving enzyme 1 (BACE1) and monoamineoxidase-B (MAO-B) enzymes in a dose-dependent manner. The saponin inhibited amyloid beta (Aβ_42_) fibrillization up to 68% at a 40 µM concentration [76]. Likewise, the anti-hyperphosphorylation and anti-neuroinflammatory effects of theasaponin E1 isolated from green tea was studied using neuroblastoma (SHY-5Y) and glioblastoma (HTB2) cells [77]. Theasaponin E1 was found to inhibit tau hyperphosphorylation and declined Aβ levels. The effects were mediated via suppression of GSK3, MAPK, CDK5, EPOE4 (E4), CAMll and PICALM expression, whereas PP1, PP2A and TREM2 expression were increased. Western blots indicated that APP, Aβ and p-tau were considerably reduced in teasaponin-E1-treated cells whereas inflammatory responses were suppressed via the inhibition of the Nf-kB pathway in a concentration-dependent manner [77]. 

### 5.1. Brief Chemistry of Saponins

Saponins are composed of an aglycone and carbohydrate moieties. The aglycone part can be a steroid or a triterpene and usually has various substituents such as -CH_3_, -H, -COOH. The structural diversity among saponins is attributed to variation in the number and type of carbohydrate groups. In majority of the cases, the saponins contain various carbohydrates such as hexoses (glucose and galactose), pentoses (xylose, arabinose) and uronic acids. Sometime the carbohydrates contain amino functionalities, as in the case of glucosamine [78]. Based on the type of aglycone, saponins are grouped as triterpenoid glycosides, steroidal glycosides and alkaloid glycosides. Triterpenes consist of three monoterpenes (each 10 carbon atoms). Based on the number of glycone moieties, the resultant saponins are either bidesmosidic or monodesmosidic terpernoid saponins. For instance, when a triterpene aglycone combines with two carbohydrate moieties, they result in the formation of bidesmosidic triterpenoid saponins, whereas the combination of a triterpene with one carbohydrate moiety results in the formation of a monodesmosidic triterpenoid saponin. Further, a steroidal aglycone, when combined with a carbohydrate moiety, results in the formation of a steroidal saponin. Similarly, when the alkaloid aglycone is combined with a sugar moiety, it forms an alkaloid saponin [79]. 

### 5.2. Occurrence and Distribution in Plants

Various plant families synthesize and store saponins [78,80,81]. These are present both in domestic as well as in wild plants. In cultivated crops, the predominant saponins are triterpenoids. Some of the plant families that contain saponins are amaranthaceae, aquifoliaceae, apiaceae, berberidaceae, chenopodiaceae, cucurbitaceae, caryophyllaceae, zygophyllaceae, leguminosae and myrsinaceae, along with some other families [81,82,83]. Legumes, including beans, peas and soybeans, are rich in triterpenoid saponins. The grasses and cereals are saponin-deficient, except for the species in Avena (Oas), which are rich in steroidal and triterpenoid saponins [84]. Some families of the plants that contain steroidal saponins include alliaceae, agavaceae, asparagaceae, amaryllidaceae, scrophulariaceae, bromeliaceae, palmae, liliaceae and dioscoreaceae. They are also stored in crop plants such as alliums, yam, asparagus, ginseng, yucca and fenugreek [85,86]. The tubers of *Dioscorea villosa* (wild yam) have saponin dioscin in abundance, which upon hydrolysis, gives diosgenin, a steroidal aglycone, which is used as a precursor for steroid synthesis, including pregnenolone, progesterone and cortisone commercially [87,88]. Some members of the family Solanaceae such as potato, tomato, capsicum and aubergine have glyco-alkaloids [89,90]. Avenacins, which are triterpenoid saponins having antifungal activities, are synthesized and then are released ffrom the roots of oat [90,91,92].

## 6. Neuropharmacological Potentials of Saponins

**Figure 2:**
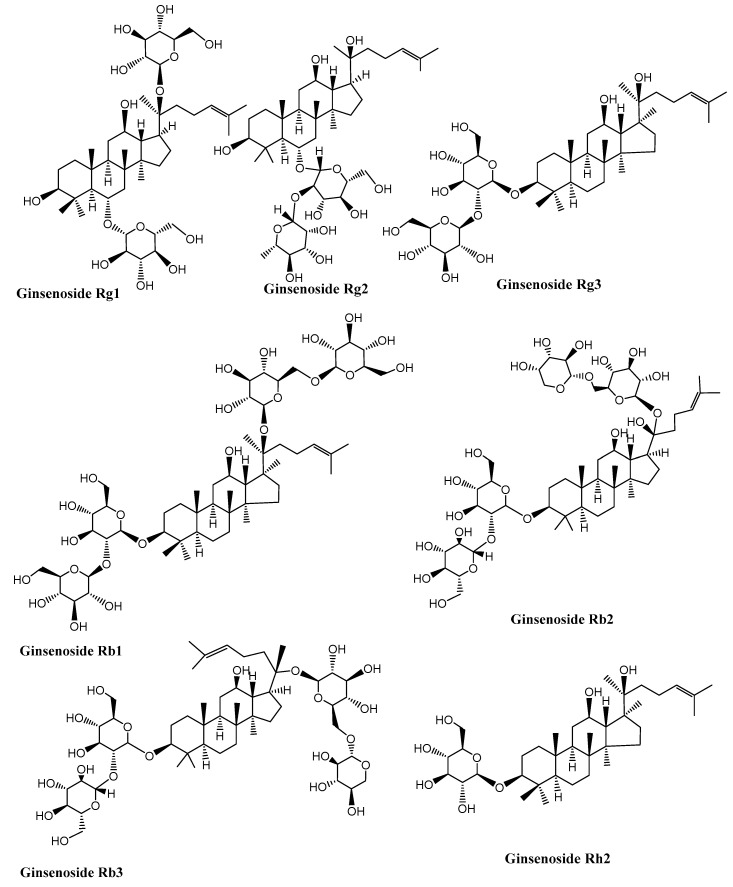
Structures of some ginseng saponins having neuroprotective effects. (Chemical structures were generated using ChemBioDraw ultra version 14).

**Figure 3:**
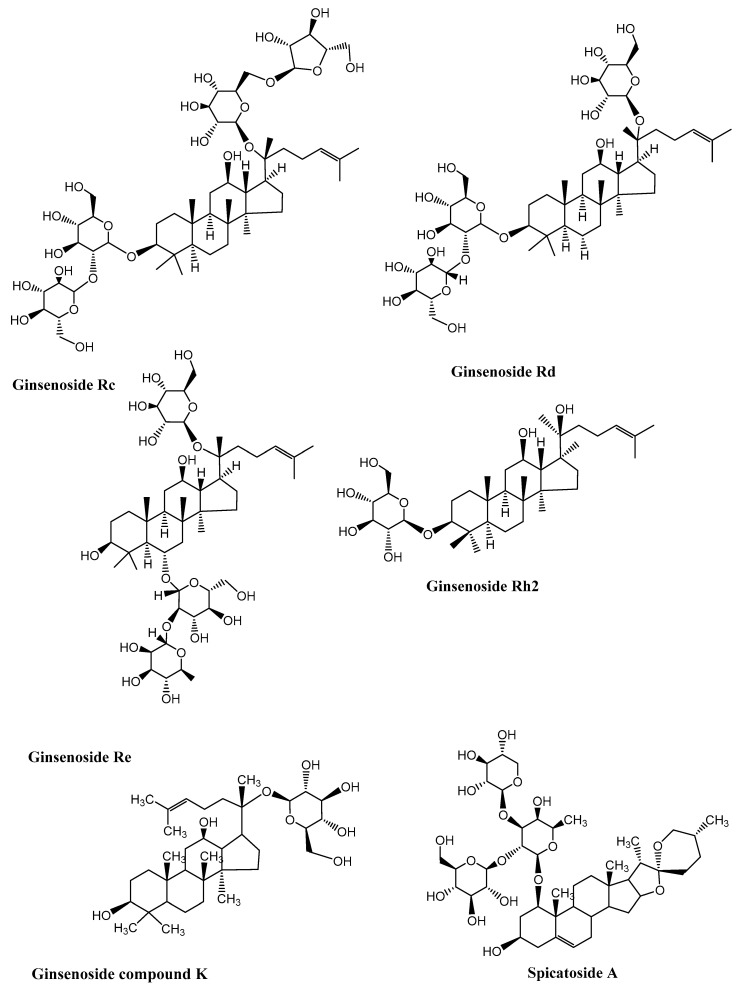
Chemical structure of neuroprotective saponins from ginseng. (Chemical structures were generated using ChemBioDraw ultra version 14).

**Figure 4:**
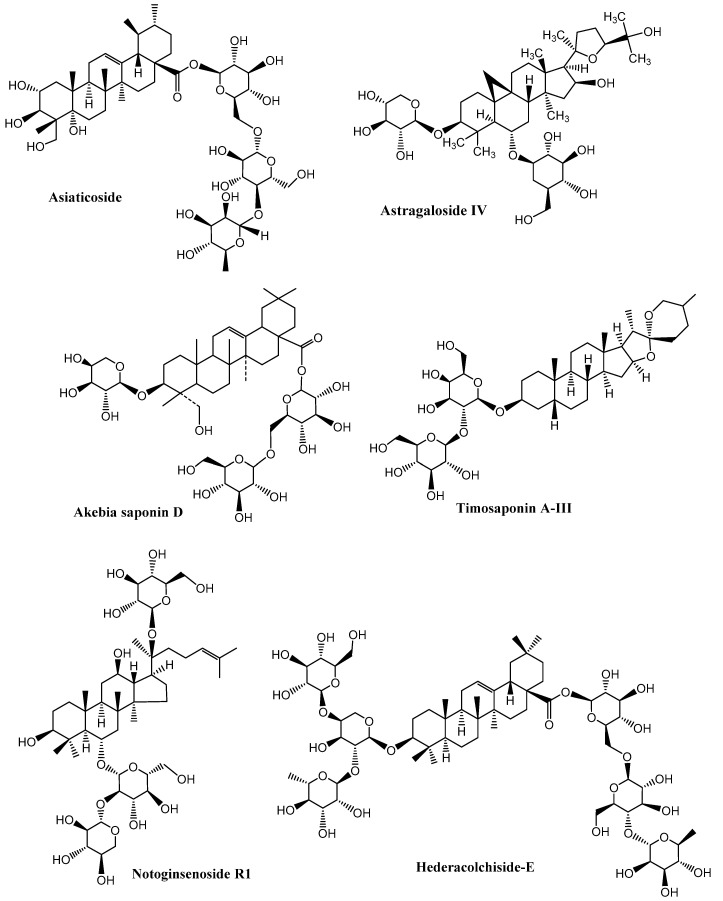
Neuroprotective saponins from medicinal plants. (Chemical structures were generated using ChemBioDraw ultra version 14).

**Figure 5:**
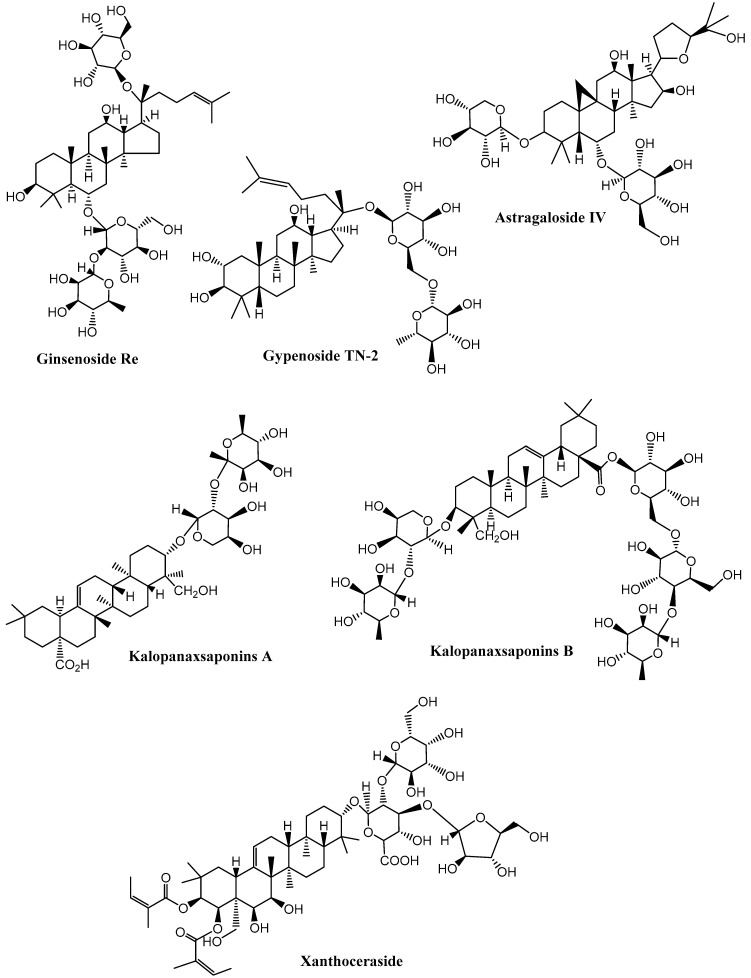
Neuroprotective saponins from plants. (Chemical structures were generated using ChemBioDraw ultra version 14).

Neurodegenerative diseases are a combination of diseases having wide-spread etiologies as well as clinical symptoms that include AD, PD and HD. Various saponins (Figure 2, Figure 3, Figure 4 and Figure 5) have proven efficacy against several neurodegenerative disorders including AD. 

### 6.1. Dementia and Age-Related Cognitive Decline

The cognitive performance is slowly and progressively lost during the aging of the brain. Sufficient pre-clinical data are available indicating that saponins have potential efficacy against various pathological targets of AD. Yet, the clinical evidence regarding their efficacy, target bioavailability and disposition in the body is limited. In mice, it has been demonstrated that ginsenoside compound K, generated by the intestinal bacteria and that is a protopanaxadiol-type saponin metabolite, shows prominent recovery from axonal atrophy, memory impairment, and synaptic loss. Furthermore, the effect produced by ginsenoside K on the axonal reconstruction was verified in the cultured cortical neurons [93]. Rb1 and Rg1 ginsenoside could enhance neural plasticity; particularly the Rg1, which is one small molecular reagent, could enhance the differentiation as well as the proliferation rate of the neural progenitor cells present in the hippocampal dentate gyrus in normal adult mice, as well as the global ischemia model in gerbils. This provides valuable data to treat AD and some other neurodegenerative disorders that involve the loss of neurons [94]. One of the main pathological characteristics of AD is the development of neurotoxic β-amyloid protein (Aβ). In rats with Aβ-induced dementia, timosaponins could increase memory and learning capacity remarkably. The ginsenosides Re, Rg1, Rg3 and ginseng produced substantial decline in the Aβ amount observed in the animals’ brains after a single dose of these agents administered orally. These results shows that ginseng, as well as the purified ginsenosides, might show similar useful effects [95]. Hederacolchiside-E, which is an oleanolic glycoside when given orally at 30 and 60 mg kg^−1^ doses, shows an increase in the step-through latency time as efficiently as tacrine given at a 30 mg kg^−1^ oral dose using the passive avoidance paradigm [96]. The total saponin content of *Dipsacus asper* has also been reported to offer protection against Aβ-induced neuronal toxicity [97]. In the cultured cortical neurons, ginsenoside Rb1 attenuates Aβ-induced toxicity in a dose-dependent manner [98]. Ginsenoside Rg3 can cause Aβ internalization, intake, as well as digestion and hence has been found to be therapeutically useful for the prevention of AD. A saponin part from *D. asper*, Akebia saponin D, offers protection of PC12 cells (a cell line that is gained from a pheochromocytoma of the adrenal medulla of rats) against cytotoxicity induced by Aβ. Akebia saponin D may meliorate the memory- and learning-related issues induced by nucleus basalis magnocellularis injury. It can protect neurons and clearly increase the level of ACh as well as ChAT activity and thus decreases AChE activity [99] (Table 1, Figure 2). 

### 6.2. Anti-Amyloid and Anti-Neurofibrillay Tangles Potential of Saponins

**Figure 6:**
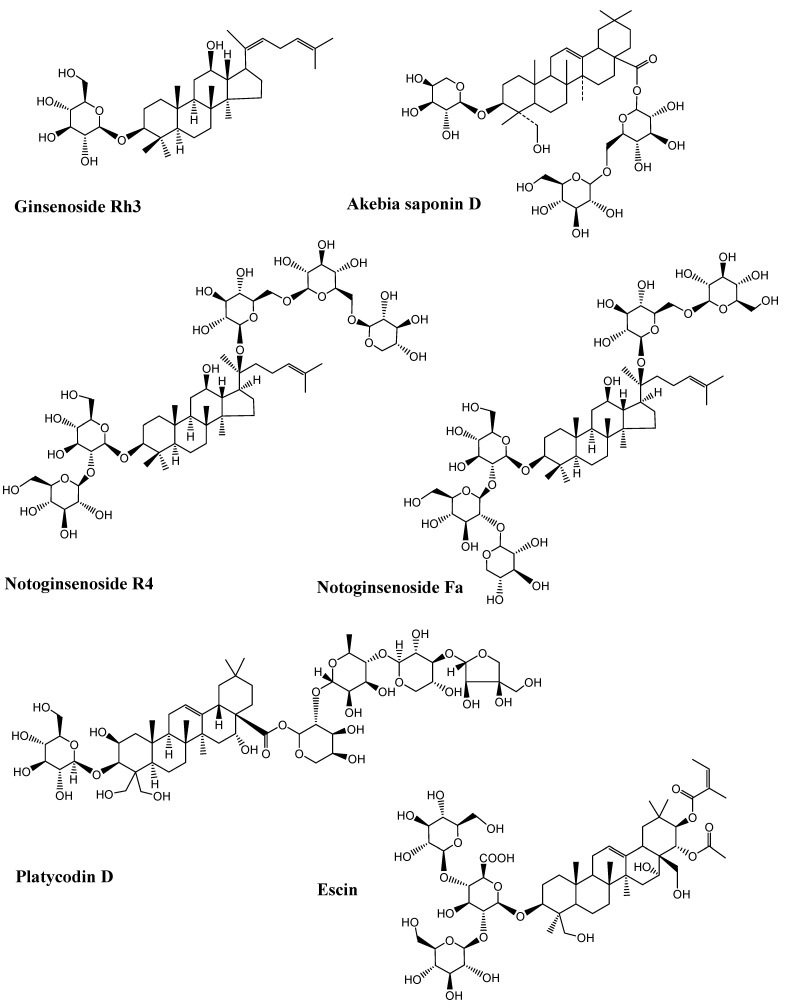
Chemical structure of bioactive saponins discussed in the study (Chemical structures were generated using ChemBioDraw ultra version 14).

In a study, the aqueous extracts from the *Asparagus racemosus* and its active metabolite sarsasapogenin was tested against various pathological targets of AD. Sarsasapogenin (SRS) is a steroidal saponin that is naturally present in medicinal plants and is widely used as a starting material for synthetic steroids in drug industry. Various steroidal saponins such as protodiosin, diosin, and shatavarin (I-VIII) sapogenins, which include sarsapogenin, are abundant in *A. racemosus* (Figure 6). Enzyme BACE-1 is responsible mainly for producing APP miscleaved fragments, which in the amyloidogenic pathway of AD makes Aβ peptide, either of 40 or 42 residues. The Aβ peptide is a 4KDa fragment which has the characteristics of self-aggregation and forms a toxic Aβ_42_ protofibril, which is the main pathological factor in AD. The *A. racemosus* root extract and SRS were studied to determine its anti-amyloidogenic activity, by inhibiting the aggregation of Aβ_42_ or reverse its oligomerization to treat AD. For the analysis of SRS anti-amyloidogenic characteristics, a Thioflavin (ThT) flouresence assay was used along with the red shift evaluation in a CR (Congo Red) dye binding assay [76]. As ThT binds to Aβ_42_ protofibrils it brightly fluoresces at 480 nm wavelength (emission), while at the same excitation wavelength of 450 nm, free ThT quenches. Hence it is used widely as a probe to quantify the formation of amyloid fibrils. It was found that SRS inhibits the Aβ_42_ fibril formation significantly in a concentration-dependent way (11–44 μM), showing 52% florescence inhibition at a concentration of 44 μM, in comparison with the control (only Aβ_42_). In this study, tannic acid was used as a positive control that showed 85% Aβ_42_ aggregation inhibition. The SRS anti-amyloidogenic effects on the formation of Aβ_42_ was further complemented by morphological analyzing of Aβ_42_ aggregate via TEM. After the 48 h’ completion of the process of aggregation, various long, branched and dense fibrils were found in Aβ_42_ controls, which showed the property of amyloid fibrils, while in samples of Aβ_42_ treated with SRS, there were shorter, scattered and fewer Aβ_42_ fibrils. It was concluded from this study that by acting on Aβ_42_ and BACE1, SRS could be used as MTDL (multi-target directed ligands) to relieve AD symptoms [76].

The plant of green tea (*Camellia sinensis*) is a rich source of various bioactive metabolites including saponins. In a study, pure theasaponin E1 was isolated from the seeds of the green tea plant and was tested against anti-neurodegenerative potentials in AD models [77]. It was found that theasaponin E1 significantly reduces tau phosphorylation. It produces this effect by suppressing as well as reducing the gene expression and different kinase activity that is responsible for tau protein hyperphosphorylation, which contributes to the formation and aggregation of NFTs and is linked with the production of Aβ and the pathogenesis of AD. In this study, cells of SHY-5Y neuroblastomas were used to determine the theasaponin E1 inhibitory activity on tau phosphorylation through inhibition or suppression of the level of expression as well as the activities of different kinases involved in this procedure. Theasaponin E1 was found to decrease the level of gene expression in SHY-5Y neuroblastoma and the in vitro activity of different kinases such as CDK5, GS3β, ERK and CAMII in a dose-dependent manner [77].

### 6.3. Efficacy in Parkinsonism

Parkinson’s disease (PD) is caused by the dopaminergic neuronal loss in the SN pars compacta. The saponin panaxatriol renders neuroprotection against behavioral impairments and dopaminergic neuronal loss induced by the neurotoxic compound 1-methyl-4-phenyl-1,2,3,6-tetrahydropyridine (MPTP). MPTP is commonly used for the development of PD in experimental models, via suppressing the over-expression of cyclooxygenase-2, enhancing the tyrosine hydroxylase and suppressing mitochondria-mediated apoptosis [100]. Ginsenoside Re helps in the dopaminergic neuron protection from MPTP-induced apoptosis via the expression of Bcl mRNA, elevating Bcl-2 protein (an apoptotic protein) levels, lowering the Bax protein (a pro-apoptotic protein) levels, the expression of Bax mRNA and inhibiting the activation of caspase-3 in the PD mouse model [101].

In PD patients, the elevated level of iron in brain SN causes neuronal death, which involves the down-regulation of iron transporters such as FP1 and DMT1. Ginsenoside Rg1 in pre-treated mice increases the level of dopamine as well as the contents of its metabolite in the striatum and also increases the expression of tyrosine hydroxylase in the SN. Hence it attenuates the MPTP-elevated levels of iron, reduces the expression of DMT1 and increases the expression of FP1 in the SN [102]. Madecassosides have been reported to exhibit neuroprotective activity in MPTP-induced Parkinsonism models and could retrieve the DA (dopamine) depletion and enhance the Bcl-2/Bax ratio and BDNF protein expression [103].

**Table 1:** Sources, bioactives and underlying neuroprotective mechanisms of saponins.

S. no	Botanical Name	Compounds	Disease/Model	Neuroprotective Mechanism	Ref.
1	*Gynostemma pentaphyllum*	Gypenoside TN-2	Learning deficit	Brain-derived neurotrophic factor (BDNF) and, cAMP-response element binding protein (CREB)	[104]
2	*Liriope platyphylla*	Spicatoside A	AD	Nerve growth factor (NGF), extracellular signal-regulated kinase (ERK), PI3-kinase/serine/threonine kinase (AKT), Cell surface transmembrane receptor tyrosine kinase (TrkA) receptor activation, neural networks reconstruction	[105]
3	*Anemarrhena asphodeloides*	Timosaponin AIII	AD	Acetylcholinesterase (AChE) Inhibition	[106]
4	*Xanthoceras sorbifolia*	Xanthoceraside	AD	Acetylcholinesterase (AChE) inhibition, antioxidant	[107]
5	*Polygala tenuifolia*	Onjisaponin F	AD	Nerve growth factor (NGF), Choline Acetyltransferase (ChAT)	[108]
6	*Kalopanax pictus*	Kalopanaxsaponins A	AD	Acetylcholinesterase (AChE) inhibition, cAMP-response element binding protein (p-CREB)	[109]
7	*K. pictus*	Kalopanaxsaponins B	AD	Acetylcholinesterase (AChE) inhibition, cAMP-response element, binding protein (p-CREB)	[109]
8	*Astragalus membranaceus*	Astragaloside IV	PD, Stroke	Antioxidant, tumor interleukin-1β (IL-1β), necrosis factor α (TNFα), Nuclear factor kappa B (NF-κB), Ca^2+^ influx, regeneration of the neural network	[74]
9	*Dipsacus asper*	Akebia saponin D	AD	mitogen-activated protein kinase (MAPK) anti- apoptosis	[99]
10	*Panax ginseng*	Ginsenoside Rb1	AD, Stroke, HD	Antioxidant, Interleukin 6 (IL-6), necrosis factor α (TNF-α), anti-apoptosis, Ca^2+^ influx, Nerve growth factor (NGF), Glial Cell-Line Derived Neurotrophic Factor (GDNF), Brain-derived neurotrophic factor (BDNF), tau phosphorylation, NF-κB, PKA, Gβ1/PI3K/Akt, Ho-1, neurite outgrowth enhancing, TNF-α, NF-Κb	[74]
11	*P. ginseng*	Ginsenoside Rg1	PD, AD, Stroke	Antioxidant, Tumor necrosis factor α (TNF-*α)*, NO, BDNF, GDNF, IGF-IR, NGF, Nuclear factor kappa B (NF-κB), PKA, JNK, ER, AChE, signaling pathway, neural networks reconstruction	[74]
12	*P. ginseng*	Ginsenoside Rg3	AD	Antioxidant, Tumor necrosis factor α (TNF-*α),* promote A*β* intake, iNOS, NMDA, interleukin-1β (IL-1β), AP-1, MSRA, PKA,	[74]
13	*P. ginseng*	Ginsenoside Rh2	AD	Tumor necrosis factor α (TNF-*α)*, NMDA, AP-1, JNK-AP-1, PKA	[110]
14	*P. ginseng*	Ginsenoside compound K	AD	Nuclear factor kappa B (NF-κB), Tumor necrosis factor α (TNF-α), interleukin-1β (IL-1β), GABA, iNOS, Intercellular adhesion molecule-1 (ICAM-1), JNK/activator protein-1 (AP-1)-signaling pathway (JNK-AP-1),	[111]
15	*P. ginseng*	Ginsenoside Re	AD	Inhibit BACE1 via activation of PPARγ, and reduce the generation of Aβ_1–40_ and Aβ_1–42_	[112]
16	*P. ginseng*	Ginsenoside Rd	Stroke	Antioxidant, iNOS, cyclooxigenase-2 (COX-2), prostaglandins E2 (PGE_2_), Ca^2+^ influx, tau phosphorylation	[113,114]
17	*P. ginseng*	Ginsenoside Rg2	Stroke	Anti-apoptosis	[115,116]
18	*P. ginseng*	Ginsenoside Rh3	Microglia cells	iNOS, TNF-*α*, IL-1*β*	[117]
19	L. macranthoides	Akebiasaponin D	AD	antagonizes Aβ25-35-induced cytotoxicity in PC 12 cells	[118]
20	*Panax notoginseng*	Notoginsenoside R1	Neuroprotection	NMDA, Bcl-2/Bax, Ca^2+^ influx	[119]
21	*P. notoginseng*	Notoginsenoside R4	Neurite growth	Neural networks reconstruction	[120]
22	*P. notoginseng*	Notoginsenoside Fa	Neurite growth	Neural networks reconstruction	[120]
23	*Platycodon grandiflorum*	Platycodin D	Stroke	NF-*κ*B, COX-2	[121]
24	*P. grandiflorum*	2″-*o*-Acetyl-polygalacin D2	Stroke	NF-*κ*B, COX-2	[121]
25	*White ginseng*	Extract	AD	AChE/BChE	[122]
26	Red ginseng	Extract	AD	AChE/BChE	[122]
27	Black ginseng	Extract	AD	AChE/BChE, antioxidant	[122]
28	*P. ginseng*	Ginsenoside Rb3	Neurite growth	Antioxidant, GABA receptor, neurite outgrowth enhancing	[123]
29	*P. ginseng*	Ginsenoside Rc	HD	Ca^2+^ signaling pathway	[124]
30	*P. ginseng*	Ginsenoside Rd	Stroke	Antioxidant, iNOS, COX-2, PGE_2,_ Ca^2+^ influx, tau phosphorylation	[113,114]
31	*Asparagus racemosus*	Sarsasapogenin	AD	Inhibits AChE/BChE, MAO-B, Inhibits Aβ_42_) fibrillization	[76]
32	*Green Tea*	theasaponin E1	AD	Inhibits tau hyperphosphorylation, declined Aβ levels, reduce inflammation	[77]
33	*Panax notoginseng*	Notoginsenoside R1	Neuronal cells	B-cell lymphoma protein 2 (Bcl-2)-associated X (Bax), N-methyl-D-aspartate (NMDA) receptors, Ca^2+^ influx	[119]
34	*C. asiatica*	Asiaticoside	PD	Modulation of B-cell lymphoma protein *2* (*Bcl*-*2*)-associated X (*Bax*), free radicals, dopamine balance	[125]
35	*Astragalus membranaceus*	Astragaloside IV	PD, Stroke,	Suppression of free radicals, TNF-α, NF-κB, IL-1β, Ca^2+^ influx, Improvement in regeneration of the neural network	[74]

## 7. Underlying Neuroprotective Mechanisms of Saponins

### 7.1. Mechanism Mediated through Antioxidant Activity

**Figure 7:**
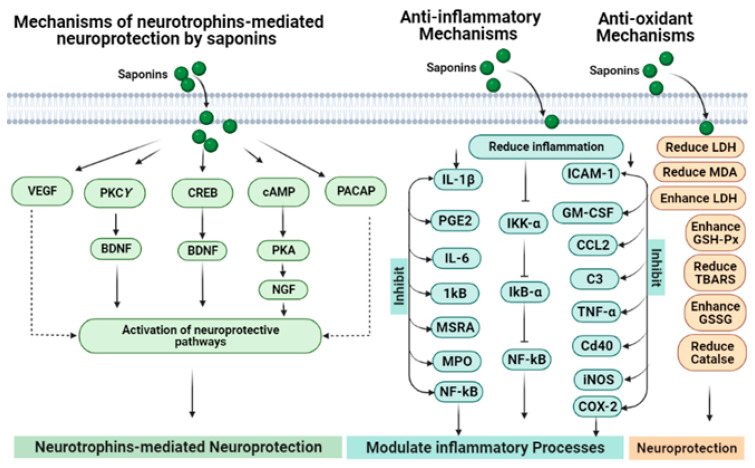
Involvement of neurotrophic modulation, anti-inflammation and anti-oxidant mechanisms in the neuroprotective potentials of saponins.

The antioxidant potentials of saponins are linked with the neuroprotective effects of these compounds (Figure 7). The oxidative stress produced by pre-oxidants in neurons and neural cell lines can be counteracted by saponins via their anti-radical effects [113,123,126,127,128]. Ginsenoside Rd reduces H_2_O_2_-mediated oxidative stress using PC1_2_ cells [113]. When Ginsenoside Rd is used using oxygen–glucose deprivation and reoxygenation models, it shows significant neuroprotection [114]. It has been determined that antioxidant effects of these compounds are mediated via an increase in glutathione and decline in intracellular ROS and MDA production. They also increase the level of indigenous antioxidant enzymes including catalase, SOD, and GSP-Px and can also reduce the lipid peroxidation and block the oxidative pathways [98]. Ginsenosides enhance the antioxidant potentials as well as upregulate the neuronal plasticity-associated proteins such as BDNF, p-CREB, PSD-95, phospho-calcium–calmodulin-dependent kinase II, phosphor-*N*-methyl-D-aspartate receptor 1, phosphor-PKA catalytic *β* subunit and PKC*γ* subunit in the hippocampus regions, which might be the mechanisms to the prevention of memory loss in animal models [118]. Ginsenosides Rb1 and Rb3 exhibit neuroprotection in glutamate-treated cortical cells. These ginsenosides stop excessive NO production that causes glutamate neurotoxicity regularly, maintains the SOD level and also diminishes the MDA formation and calcium influx. It has been determined that ginsenoside Rb1 can protect A*β*-induced increases in the LDH release, SOD activity and MDA production in neurons [123,128]. Asiaticoside was found to be beneficial in MPTP-induced Parkinsonism due to its neuroprotective potential that includes its antioxidant activity, by regulating the dopamine metabolic balance and enhancing the Bcl-2/Bax ratio [125].

### 7.2. Mechanism Linked to Modulation of Neurotransmitters

Saponins could produce neuroprotection by modulating neurotransmitters. Saponins can produce nervous system protection by regulating ACh and dopamine levels through modulation of adenosine or NMDA receptors.

### 7.3. Modulation of NMDA Receptors

The neurotransmitter glutamate normally acts on NMDA receptors, which consist of the NR1/NR2B subunit assembly in the brain. NMDA is a derivative of an amino acid that serves as an NMDA-receptor-specific agonist. The excitotoxicity of glutamate is considered as a condition in which increased glutamate causes the degeneration and dysfunction of neurons. The notoginsenoside R1 specifically helps to protect the neurons from glutamate excitotoxicity and is mediated by NMDA receptors. In addition to preventing decreased Bcl-2 and increased Bax expression levels, it has been determined that notoginsenoside R1 can protect the neurons from excitotoxicity, overproduction of intracellular reactive oxygen species, increased intracellular free Ca^2+^, and mitochondrial membrane depolarization potential in cultured neurons that is promoted by glutamate [119].

### 7.4. Modulating Adenosine Receptors

Ginsenosides can block the KA-induced synaptosomal oxidative stress that is linked with hippocampal degeneration by activating adenosine A2A receptors [129]. Additionally, by activating the adenosine receptor, ginsenosides can attenuate changes in behavior and increase the binding activity of AP-1 DNA, Fos-related antigen immunoreactivity, as well as the expression of proenkephalin genes induced by methamphetamine in mouse striatum [130].

### 7.5. Saponins Reduce Tau Phosphorylation

**Figure 8:**
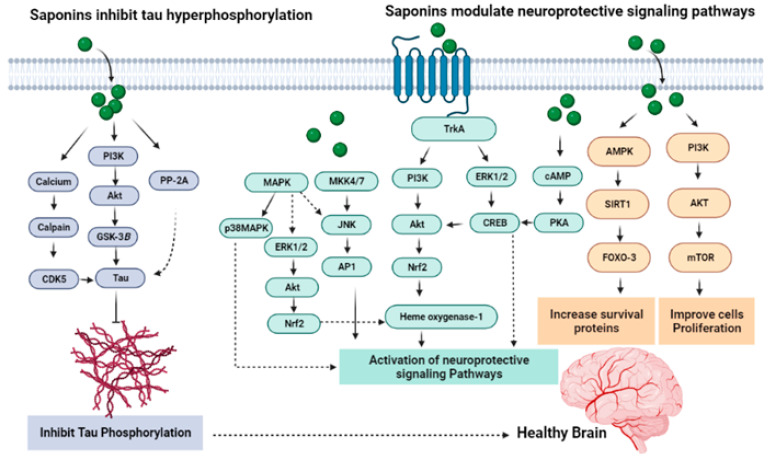
Mechanisms underlying the anti-tau process and modulation of neuroprotective signaling pathways.

Okadaic acid (OA), being an inhibitor of protein phosphatase, causes tau hyperphosphorylation in the CNS. By preventing hyperphosphorylation of tau protein, the ginsenoside Rd produced significant neuroprotection against OA-induced neurotoxicity (Figure 8). Both in vivo and in vitro activity have determined that pretreatment with ginsenoside Rd decreases OA-induced inactivation of PP-2A dramatically, which shows that the regulating activity of ginsenoside Rd on PP-2A activity has a significant role in the anti-OA-induced hyperphosphorylation process of tau proteins [130]. In neurons, abundant tau protein interacts with tubulin for stabilization of microtubules and promoting tubulin assembly into the microtubules. Tau protein hyperphosphorylation can be considered to be one of the factors in AD pathogenesis. Ginsenoside Rb1 attenuates A*β*_1–42_-induced neurotoxicity significantly in a dose-dependent way. The mechanism involved in the neuroprotection of ginsenoside Rb1 may be the blocking of the abnormal hyperphosphorylation of tau and increasing the expression of phospho-Ser133–CREB through PI3K/Akt/GSK-3*β* [98] (Figure 8).

## 8. Conclusions

Pre-clinical neuroprotective studies on saponins exhibited significant importance in the attenuation of neurodegenerative disorders. Through various mechanisms, saponins produce neuroprotective effects including antioxidant effects, modulation of neurotransmitters and through inhibition of tau phosphorylation. At present, knowledge about the molecular mechanisms of the neuroprotective effect of saponins is still partially understood. In-depth knowledge is required for the application of approaches in rational drug design, for saponin screening or for chemical syntheses of derivatives of saponins having great medicinal efficiency. Clinical studies are required on the high-potential saponins regarding their in vivo safety, bioavailability, efficacy, BBB-crossing capacity, tissue specificity and clearance from the body. Biosynthesized nano-formulation might also help in achieving target specific outcomes. 

## Data Availability

Not applicable.

## References

[1] Levey A.I. (2021). Progress with treatments for Alzheimer’s disease. N. Engl. J. Med..

[2] Jahn H. (2013). Memory loss in Alzheimer’s disease. Dialogues Clin. Neurosci..

[3] Liu Z., Zhang A., Sun H., Han Y., Kong L., Wang X. (2017). Two decades of new drug discovery and development for Alzheimer’s disease. RSC Adv..

[4] Ju Y., Tam K.Y. (2022). Pathological mechanisms and therapeutic strategies for Alzheimer’s disease. Neural Regen. Res..

[5] Ayaz M., Nawaz A., Naz F., Ullah F., Sadiq A., Islam Z.U. (2022). Phytochemicals-based therapeutics against Alzheimer’s disease: An update. Curr. Top. Med. Chem..

[6] Pinyopornpanish K., Soontornpun A., Wongpakaran T., Wongpakaran N., Tanprawate S., Pinyopornpanish K., Nadsasarn A., Pinyopornpanish M. (2022). Impact of behavioral and psychological symptoms of Alzheimer’s disease on caregiver outcomes. Sci. Rep..

[7] Ernst R.L., Hay J.W. (1997). Economic research on Alzheimer disease: A review of the literature. Alzheimer Dis. Assoc. Disord..

[8] Karthika C., Appu A.P., Akter R., Rahman M.H., Tagde P., Ashraf G.M., Abdel-Daim M.M., Hassan S.S.u., Abid A., Bungau S. (2022). Potential innovation against Alzheimer’s disorder: A tricomponent combination of natural antioxidants (vitamin E, quercetin, and basil oil) and the development of its intranasal delivery. Environ. Sci. Pollut. Res..

[9] Qiu C., Kivipelto M., Von Strauss E. (2009). Epidemiology of Alzheimer’s disease: Occurrence, determinants, and strategies toward intervention. Dialogues Clin. Neurosci..

[10] Alzheimer’s Association (2020). Alzheimer’s disease facts and figures. Alzheimers Dement..

[11] Conrado D.J., Duvvuri S., Geerts H., Burton J., Biesdorf C., Ahamadi M., Macha S., Hather G., Francisco Morales J., Podichetty J. (2020). Challenges in Alzheimer’s Disease Drug Discovery and Development: The role of modeling, simulation, and open data. Clin. Pharmacol. Ther..

[12] Hebert L.E., Weuve J., Scherr P.A., Evans D.A. (2013). Alzheimer disease in the United States (2010–2050) estimated using the 2010 census. Neurology.

[13] Evans D.A. (1990). Estimated prevalence of Alzheimer’s disease in the United States. Milbank Q..

[14] Yadav M., Pandey P., Sharma P. (2022). Understanding the genetic, molecular, and cellular basis of ageing as the biggest risk factor of Alzheimer’s disease. Eur. J. Biol. Res..

[15] Brookmeyer R., Gray S., Kawas C. (1998). Projections of Alzheimer’s disease in the United States and the public health impact of delaying disease onset. Am. J. Public Health.

[16] Menzin J., Lang K., Friedman M., Neumann P., Cummings J.L. (1999). The economic cost of Alzheimer’s disease and related dementias to the California Medicaid program (“Medi-Cal”) in 1995. Am. J. Geriatr. Psychiatry.

[17] Omura J.D., McGuire L.C., Patel R., Baumgart M., Lamb R., Jeffers E.M., Olivari B.S., Croft J.B., Thomas C.W., Hacker K. (2022). Modifiable risk factors for Alzheimer disease and related dementias among adults aged ≥45 years—United States, 2019. Morb. Mortal. Wkly. Rep..

[18] Morrison A.S., Lyketsos C. (2005). The pathophysiology of Alzheimer’s disease and directions in treatment. Adv. Stud. Nurs..

[19] Förstl H., Zerfaß R., Geiger-Kabisch C., Sattel H., Besthorn C., Hentschel F. (1995). Brain atrophy in normal ageing and Alzheimer’s disease: Volumetric discrimination and clinical correlations. Br. J. Psychiatry.

[20] Hippius H., Neundörfer G. (2003). The discovery of Alzheimer’s disease. Dialogues Clin. Neurosci..

[21] Bianchetti A., Ranieri P., Margiotta A., Trabucchi M. (2006). Pharmacological treatment of Alzheimer’s Disease. Aging Clin. Exp. Res..

[22] Ovais M., Zia N., Ahmad I., Khalil A.T., Raza A., Ayaz M., Sadiq A., Ullah F., Shinwari Z.K. (2018). Phyto-Therapeutic and Nanomedicinal Approach to Cure Alzheimer Disease: Present Status and Future Opportunities. Front. Aging Neurosci..

[23] Chen J.X., Yan S.D. (2007). Amyloid-β-induced mitochondrial dysfunction. J. Alzheimer’s Dis..

[24] Wang X., Su B., Perry G., Smith M.A., Zhu X. (2007). Insights into amyloid-β-induced mitochondrial dysfunction in Alzheimer disease. Free Radic. Biol. Med..

[25] Tong X., Li X., Ayaz M., Ullah F., Sadiq A., Ovais M., Shahid M., Khayrullin M., Hazrat A. (2020). Neuroprotective studies on *Polygonum hydropiper* L. essential oils using transgenic animal models. Front. Pharmacol..

[26] Ayaz M., Sadiq A., Junaid M., Ullah F., Subhan F., Ahmed J. (2017). Neuroprotective and anti-aging potentials of essential oils from aromatic and medicinal plants. Front. Aging Neurosci..

[27] Ayaz M., Sadiq A., Junaid M., Ullah F., Ovais M., Ullah I., Ahmed J., Shahid M. (2019). Flavonoids as prospective neuroprotectants and their therapeutic propensity in aging associated neurological disorders. Front. Aging Neurosci..

[28] Suzuki N., Cheung T.T., Cai X.-D., Odaka A., Otvos L., Eckman C., Golde T.E., Younkin S.G. (1994). An increased percentage of long amyloid β protein secreted by familial amyloid β protein precursor (βAPP717) mutants. Science.

[29] Rong X., Jiang L., Qu M., Hassan S.S.u., Liu Z. (2020). Enhancing Therapeutic Efficacy of Donepezil by Combined Therapy: A Comprehensive Review. Curr. Pharm. Des..

[30] Kaur D., Behl T., Sehgal A., Singh S., Sharma N., Badavath V.N., Hassan S.S.u., Hasan M.M., Bhatia S., Al-Harassi A. (2022). Unravelling the potential neuroprotective facets of erythropoietin for the treatment of Alzheimer’s disease. Metab. Brain Dis..

[31] Brion J.-P. (1998). Neurofibrillary tangles and Alzheimer’s disease. Eur. Neurol..

[32] Amber S., Zahid S., Malik N. (2022). Rosmarinus officinalis and Methylphenidate Exposure Improves Cognition and Depression and Regulates Anxiety like Behavior in AlCl3-induced Mouse Model of Alzheimer’s Disease. Front. Pharmacol..

[33] Perry E.K., Tomlinson B.E., Blessed G., Bergmann K., Gibson P.H., Perry R.H. (1978). Correlation of cholinergic abnormalities with senile plaques and mental test scores in senile dementia. Br. Med. J..

[34] Mir N.T., Saleem U., Anwar F., Ahmad B., Ullah I., Hira S., Ismail T., Ali T., Ayaz M. (2019). Lawsonia Inermis Markedly Improves Cognitive Functions in Animal Models and Modulate Oxidative Stress Markers in the Brain. Medicina.

[35] Saleem U., Akhtar R., Anwar F., Shah M.A., Chaudary Z., Ayaz M., Ahmad B. (2021). Neuroprotective potential of Malva neglecta is mediated via down-regulation of cholinesterase and modulation of oxidative stress markers. Metab. Brain Dis..

[36] Butterfield D.A., Griffin S., Munch G., Pasinetti G.M. (2002). Amyloid β-peptide and amyloid pathology are central to the oxidative stress and inflammatory cascades under which Alzheimer’s disease brain exists. J. Alzheimer’s Dis..

[37] Zhu X., Raina A.K., Smith M.A. (1999). Cell cycle events in neurons: Proliferation or death?. Am. J. Pathol..

[38] Zhu X., Lee H.-G., Casadesus G., Avila J., Drew K., Perry G., Smith M.A. (2005). Oxidative imbalance in Alzheimer’s disease. Mol. Neurobiol..

[39] Rozemuller J., Eikelenboom P., Stam F. (1986). Role of microglia in plaque formation in senile dementia of the Alzheimer type. Virchows Arch. B.

[40] Hassan S.S.u., Abdel-Daim M.M., Behl T., Bungau S. (2022). Natural Products for Chronic Diseases: A Ray of Hope. Molecules.

[41] Rogers J., Shen Y. (2000). A perspective on inflammation in Alzheimer’s disease. Ann. N.Y. Acad. Sci..

[42] Zotova E., Nicoll J.A., Kalaria R., Holmes C., Boche D. (2010). Inflammation in Alzheimer’s disease: Relevance to pathogenesis and therapy. Alzheimers Res. Ther..

[43] Akiyama H., Barger S., Barnum S., Bradt B., Bauer J., Cole G., Cooper N., Eikelenboom P., Emmerling M., Fiebich B.L. (2000). Inflammation and Alzheimer’s disease. Neurobiol. Aging.

[44] Kalaria R.N. (1999). Microglia and Alzheimer’s disease. Curr. Opin. Hematol..

[45] Arnaud L., Robakis N.K., Figueiredo-Pereira M.E. (2006). It may take inflammation, phosphorylation and ubiquitination to ‘tangle’in Alzheimer’s disease. Neurodegener. Dis..

[46] Gorlovoy P., Larionov S., Pham T.T.H., Neumann H. (2009). Accumulation of tau induced in neurites by microglial proinflammatory mediators. FASEB J..

[47] Nicoll J.A., Mrak R.E., Graham D.I., Stewart J., Wilcock G., MacGowan S., Esiri M.M., Murray L.S., Dewar D., Love S. (2000). Association of interleukin-1 gene polymorphisms with Alzheimer’s disease. Ann. Neurol. Off. J. Am. Neurol. Assoc. Child Neurol. Soc..

[48] Hayes A., Green E., Pritchard A., Harris J., Zhang Y., Lambert J., Chartier-Harlin M., Pickering-Brown S., Lendon C.L., Mann D.M. (2004). A polymorphic variation in the interleukin 1A gene increases brain microglial cell activity in Alzheimer’s disease. J. Neurol. Neurosurg. Psychiatry.

[49] Culpan D., MacGowan S.H., Ford J.M., Nicoll J.A., Griffin W.S., Dewar D., Cairns N.J., Hughes A., Kehoe P.G., Wilcock G.K. (2003). Tumour necrosis factor-α gene polymorphisms and Alzheimer’s disease. Neurosci. Lett..

[50] Horsburgh K., McCarron M.O., White F., Nicoll J.A. (2000). The role of apolipoprotein E in Alzheimer’s disease, acute brain injury and cerebrovascular disease: Evidence of common mechanisms and utility of animal models. Neurobiol. Aging.

[51] Egensperger R., Kösel S., von Eitzen U., Graeber M.B. (1998). Microglial activation in Alzheimer disease: Association with APOE genotype. Brain Pathol..

[52] Galea E., Heneka M.T., Russo C.D., Feinstein D.L. (2003). Intrinsic regulation of brain inflammatory responses. Cell. Mol. Neurobiol..

[53] Herrmann N., Lanctôt K.L., Khan L.R. (2004). The role of norepinephrine in the behavioral and psychological symptoms of dementia. J. Neuropsychiatry Clin. Neurosci..

[54] Ayaz M., Junaid M., Ullah F., Sadiq A., Khan M.A., Ahmad W., Shah M.R., Imran M., Ahmad S. (2015). Comparative chemical profiling, cholinesterase inhibitions and anti-radicals properties of essential oils from *Polygonum hydropiper* L: A Preliminary anti-Alzheimer’s study. Lipids Health Dis..

[55] Khalil A.T., Ayaz M., Ovais M., Wadood A., Ali M., Shinwari Z.K., Maaza M. (2018). In vitro cholinesterase enzymes inhibitory potential and in silico molecular docking studies of biogenic metal oxides nanoparticles. Inorg. Nano-Met. Chem..

[56] Ayaz M., Ullah F., Sadiq A., Kim M.O., Ali T. (2019). Natural products-based drugs: Potential therapeutics against Alzheimer’s disease and other neurological disorders. Front. Pharmacol..

[57] Faheem M., Shah F.A., Khan A.U., Li S.P. (2022). Investigation of Natural Isolated Compounds for Therapeutic Potential in Streptozotocin-induced Diabetic Neuroinflammation, Neurodegeneration and Neuropathic Pain. Front. Pharmacol..

[58] Ayaz M., Ali T., Sadiq A., Ullah F., Naseer M.I. (2022). Current Trends in Medicinal Plant Research and Neurodegenerative Disorders. Front. Media SA.

[59] Ratheesh G., Tian L., Venugopal J. (2017). Role of medicinal plants in neurodegenerative diseases. Biomanuf. Rev..

[60] Yang F., Lim G.P., Begum A.N., Ubeda O.J., Simmons M.R., Ambegaokar S.S., Chen P.P., Kayed R., Glabe C.G., Frautschy S.A. (2005). Curcumin inhibits formation of amyloid β oligomers and fibrils, binds plaques, and reduces amyloid in vivo. J. Biol. Chem..

[61] McClure R., Ong H., Janve V., Barton S., Zhu M., Li B., Dawes M., Jerome W.G., Anderson A., Massion P. (2017). Aerosol delivery of curcumin reduced amyloid-β deposition and improved cognitive performance in a transgenic model of Alzheimer’s disease. J. Alzheimer’s Dis..

[62] Reddy P.H., Manczak M., Yin X., Grady M.C., Mitchell A., Tonk S., Kuruva C.S., Bhatti J.S., Kandimalla R., Vijayan M. (2018). Protective effects of Indian spice curcumin against amyloid-β in Alzheimer’s disease. J. Alzheimer’s Dis..

[63] Ono K., Yoshiike Y., Takashima A., Hasegawa K., Naiki H., Yamada M. (2003). Potent anti-amyloidogenic and fibril-destabilizing effects of polyphenols in vitro: Implications for the prevention and therapeutics of Alzheimer’s disease. J. Neurochem..

[64] Vauzour D. (2014). Effect of flavonoids on learning, memory and neurocognitive performance: Relevance and potential implications for Alzheimer’s disease pathophysiology. J. Sci. Food Agric..

[65] Choi S.-M., Kim B.C., Cho Y.-H., Choi K.-H., Chang J., Park M.-S., Kim M.-K., Cho K.-H., Kim J.-K. (2014). Effects of flavonoid compounds on β-amyloid-peptide-induced neuronal death in cultured mouse cortical neurons. Chonnam Med. J..

[66] DeKosky S.T., Scheff S.W. (1990). Synapse loss in frontal cortex biopsies in Alzheimer’s disease: Correlation with cognitive severity. Ann. Neurol. Off. J. Am. Neurol. Assoc. Child Neurol. Soc..

[67] Jackson M., Gentleman S., Lennox G., Ward L., Gray T., Randall K., Morrell K., Lowe J. (1995). The cortical neuritic pathology of Huntington’s disease. Neuropathol. Appl. Neurobiol..

[68] Hassan S.S.u., Muhammad I., Abbas S.Q., Hassan M., Majid M., Jin H.Z., Bungau S. (2021). Stress driven discovery of natural products from actinobacteria with anti-oxidant and cytotoxic activities including docking and admet properties. Int. J. Mol. Sci..

[69] Mattila P., Rinne J., Helenius H., Röyttä M. (1999). Neuritic degeneration in the hippocampus and amygdala in Parkinson’s disease in relation to Alzheimer pathology. Acta Neuropathol..

[70] Ayaz M., Junaid M., Ullah F., Sadiq A., Subhan F., Khan M.A., Ahmad W., Ali G., Imran M., Ahmad S. (2016). Molecularly characterized solvent extracts and saponins from *Polygonum hydropiper* L. show high anti-angiogenic, anti-tumor, brine shrimp, and fibroblast NIH/3T3 cell line cytotoxicity. Front. Pharmacol..

[71] Liu J., Henkel T. (2002). Traditional Chinese medicine (TCM): Are polyphenols and saponins the key ingredients triggering biological activities?. Curr. Med. Chem..

[72] Güçlü-Üstündağ Ö., Mazza G. (2007). Saponins: Properties, applications and processing. Crit. Rev. Food Sci. Nutr..

[73] Sun A., Xu X., Lin J., Cui X., Xu R. (2015). Neuroprotection by saponins. Phytother. Res..

[74] Oyeleke M.B., Owoyele B.V. (2022). Saponins and flavonoids from Bacopa floribunda plant extract exhibit antioxidant and anti-inflammatory effects on amyloid beta 1-42-induced Alzheimer’s disease in BALB/c mice. J. Ethnopharmacol..

[75] Kashyap P., Muthusamy K., Niranjan M., Trikha S., Kumar S. (2020). Sarsasapogenin: A steroidal saponin from *Asparagus racemosus* as multi target directed ligand in Alzheimer’s disease. Steroids.

[76] Khan M.I., Khan M.Z., Shin J.H., Shin T.S., Lee Y.B., Kim M.Y., Kim J.D. (2022). Neuroprotective Effects of Green Tea Seed Isolated Saponin Due to the Amelioration of Tauopathy and Alleviation of Neuroinflammation: A Therapeutic Approach to Alzheimer’s Disease. Molecules.

[77] Vincken J.-P., Heng L., de Groot A., Gruppen H. (2007). Saponins, classification and occurrence in the plant kingdom. Phytochemistry.

[78] El Aziz M., Ashour A., Melad A. (2019). A review on saponins from medicinal plants: Chemistry, isolation, and determination. J. Nanomed. Res..

[79] Dinda B., Debnath S., Mohanta B.C., Harigaya Y. (2010). Naturally occurring triterpenoid saponins. Chem. Biodivers..

[80] Sparg S., Light M., Van Staden J. (2004). Biological activities and distribution of plant saponins. J. Ethnopharmacol..

[81] Parente J.P., da Silva B.P. (2009). Bioactive complex triterpenoid saponins from the Leguminosae family. Nat. Prod. Commun..

[82] Shi J., Arunasalam K., Yeung D., Kakuda Y., Mittal G., Jiang Y. (2004). Saponins from edible legumes: Chemistry, processing, and health benefits. J. Med. Food.

[83] Osbourn A.E. (2003). Saponins in cereals. Phytochemistry.

[84] Hoffmann D. (2003). Medical Herbalism: The Science and Practice of Herbal Medicine.

[85] Hostettmann K., Marston A. (2005). Saponins.

[86] He L., Mu L., Jean J.A., Zhang L., Wu H., Zhou T., Bu H. (2022). Contributions and Challenges of Public Health Social Work Practice during the Initial 2020 COVID-19 Outbreak in China. Br. J. Soc. Work..

[87] He X., Zhu Y., Yang L., Wang Z., Wang Z., Feng J., Wen X., Cheng L., Zhu R. (2021). MgFe-LDH Nanoparticles: A Promising Leukemia Inhibitory Factor Replacement for Self-Renewal and Pluripotency Maintenance in Cultured Mouse Embryonic Stem Cells. Adv. Sci..

[88] Roddick J., Melchers G. (1985). Steroidal glycoalkaloid content of potato, tomato and their somatic hybrids. Theor. Appl. Genet..

[89] Carter J.P., Spink J., Cannon P.F., Daniels M.J., Osbourn A.E. (1999). Isolation, characterization, and avenacin sensitivity of a diverse collection of cereal-root-colonizing fungi. Appl. Environ. Microbiol..

[90] Field B., Jordán F., Osbourn A. (2006). First encounters–deployment of defence-related natural products by plants. New Phytol..

[91] Hostettmann K., Marston A. (1995). Chemistry and Pharmacology of Natural Products.

[92] Tohda C., Matsumoto N., Zou K., Meselhy M.R., Komatsu K. (2004). A β (25–35)-induced memory impairment, axonal atrophy, and synaptic loss are ameliorated by M1, a metabolite of protopanaxadiol-type saponins. Neuropsychopharmacology.

[93] Cheng Y., Shen L.H., Zhang J.T. (2005). Anti-amnestic and anti-aging effects of ginsenoside Rg1 and Rb1 and its mechanism of action. ACTA Pharmacol. Sin..

[94] Chen F., Eckman E.A., Eckman C.B. (2006). Reductions in levels of the Alzheimer’s amyloid beta peptide after oral administration of ginsenosides. FASEB J. Off. Publ. Fed. Am. Soc. Exp. Biol..

[95] Han C.-K., Choi W.R., Oh K.-B. (2007). Cognition-enhancing and neuroprotective effects of hederacolchiside-E from *Pulsatilla koreana*. Planta Med..

[96] Qian Y.-H., Liu Y., Hu H.-T., Ren H.-M., Chen X.-L., Xu J.-H. (2002). The effects of the total saponin of Dipsacus asperoides on the damage of cultured neurons induced by β-amyloid protein 25–35. Anat. Sci. Int..

[97] Zheng J., Long X., Chen H., Ji Z., Shu B., Yue R., Liao Y., Ma S., Qiao K., Liu Y. (2022). Photoclick Reaction Constructs Glutathione-Responsive Theranostic System for Anti-Tuberculosis. Front. Mol. Biosci..

[98] Yu X., Wang L.-N., Ma L., You R., Cui R., Ji D., Wu Y., Zhang C.-F., Yang Z.-L., Ji H. (2012). Akebia saponin D attenuates ibotenic acid-induced cognitive deficits and pro-apoptotic response in rats: Involvement of MAPK signal pathway. Pharmacol. Biochem. Behav..

[99] Luo F.-C., Wang S.-D., Qi L., Song J.-Y., Lv T., Bai J. (2011). Protective effect of panaxatriol saponins extracted from *Panax notoginseng* against MPTP-induced neurotoxicity in vivo. J. Ethnopharmacol..

[100] Xu B.-B., Liu C.-Q., Gao X., Zhang W.-Q., Wang S.-W., Cao Y.-L. (2005). Possible mechanisms of the protection of ginsenoside Re against MPTP-induced apoptosis in substantia nigra neurons of Parkinson’s disease mouse model. J. Asian Nat. Prod. Res..

[101] Wang J., Xu H.-M., Yang H.-D., Du X.-X., Jiang H., Xie J.-X. (2009). Rg1 reduces nigral iron levels of MPTP-treated C57BL6 mice by regulating certain iron transport proteins. Neurochem. Int..

[102] Xu C.-L., Qu R., Zhang J., Li L.-F., Ma S.-P. (2013). Neuroprotective effects of madecassoside in early stage of Parkinson’s disease induced by MPTP in rats. Fitoterapia.

[103] Hong S.-W., Yang J.-H., Joh E.-H., Kim H.J., Kim D.-H. (2011). Gypenoside TN-2 ameliorates scopolamine-induced learning deficit in mice. J. Ethnopharmacol..

[104] Hur J., Lee P., Moon E., Kang I., Kim S.-H., Oh M.S., Kim S.Y. (2009). Neurite outgrowth induced by spicatoside A, a steroidal saponin, via the tyrosine kinase A receptor pathway. Eur. J. Pharmacol..

[105] Lee B., Jung K., Kim D.-H. (2009). Timosaponin AIII, a saponin isolated from Anemarrhena asphodeloides, ameliorates learning and memory deficits in mice. Pharmacol. Biochem. Behav..

[106] Chi T.-Y., Wang L.-H., Qu C., Yang B.-Z., Ji X.-F., Wang Y., Okuyama T., Yoshihito O., Zou L.-B. (2009). Protective effects of xanthoceraside on learning and memory impairment induced by Aβ25–35 in mice. J. Asian Nat. Prod. Res..

[107] Yabe T., Tuchida H., Kiyohara H., Takeda T., Yamada H. (2003). Induction of NGF synthesis in astrocytes by onjisaponins of Polygala tenuifolia, constituents of kampo (Japanese herbal) medicine, Ninjin-yoei-to. Phytomedicine.

[108] Joh E.H., Lee I.A., Kim D.H. (2012). Kalopanaxsaponins A and B isolated from *Kalopanax pictus* ameliorate memory deficits in mice. Phytother. Res..

[109] Lee E., Kim S., Chung K.C., Choo M.-K., Kim D.-H., Nam G., Rhim H. (2006). 20 (S)-ginsenoside Rh2, a newly identified active ingredient of ginseng, inhibits NMDA receptors in cultured rat hippocampal neurons. Eur. J. Pharmacol..

[110] Chung S.C., Chen Y.F. (2020). Effect of convalescent toy image design on memory recovery in patients with Alzheimer’s disease. Indian J. Pharm. Sci..

[111] Cao G., Su P., Zhang S., Guo L., Zhang H., Liang Y., Qin C., Zhang W. (2016). Ginsenoside Re reduces Aβ production by activating PPARγ to inhibit BACE1 in N2a/APP695 cells. Eur. J. Pharmacol..

[112] Ye R., Han J., Kong X., Zhao L., Cao R., Rao Z., Zhao G. (2008). Protective effects of ginsenoside Rd on PC12 cells against hydrogen peroxide. Biol. Pharm. Bull..

[113] Ye R., Li N., Han J., Kong X., Cao R., Rao Z., Zhao G. (2009). Neuroprotective effects of ginsenoside Rd against oxygen-glucose deprivation in cultured hippocampal neurons. Neurosci. Res..

[114] Zhang G., Liu A., Zhou Y., San X., Jin T., Jin Y. (2008). Panax ginseng ginsenoside-Rg2 protects memory impairment via anti-apoptosis in a rat model with vascular dementia. J. Ethnopharmacol..

[115] Zhang Y.-F., Fan X.-J., Li X., Peng L.-L., Wang G.-H., Ke K.-F., Jiang Z.-L. (2008). Ginsenoside Rg1 protects neurons from hypoxic–ischemic injury possibly by inhibiting Ca^2+^ influx through NMDA receptors and L-type voltage-dependent Ca^2+^ channels. Eur. J. Pharmacol..

[116] Park J.-S., Park E.-M., Kim D.-H., Jung K., Jung J.-S., Lee E.-J., Hyun J.-W., Kang J.L., Kim H.-S. (2009). Anti-inflammatory mechanism of ginseng saponins in activated microglia. J. Neuroimmunol..

[117] Zhou Y.-Q., Yang Z.-L., Xu L., Li P., Hu Y.-Z. (2009). Akebia saponin D, a saponin component from Dipsacus asper Wall, protects PC 12 cells against amyloid-β induced cytotoxicity. Cell Biol. Int..

[118] Gu B., Nakamichi N., Zhang W.S., Nakamura Y., Kambe Y., Fukumori R., Takuma K., Yamada K., Takarada T., Taniura H. (2009). Possible protection by notoginsenoside R1 against glutamate neurotoxicity mediated by *N*-methyl-D-aspartate receptors composed of an NR1/NR2B subunit assembly. J. Neurosci. Res..

[119] Zou K., Zhu S., Meselhy M.R., Tohda C., Cai S., Komatsu K. (2002). Dammarane-Type Saponins from Panax j aponicus and Their Neurite Outgrowth Activity in SK-N-SH Cells. J. Nat. Prod..

[120] Choi J.H., Yoo K.-Y., Park O.K., Lee C.H., Won M.-H., Hwang I.K., Ryu S.Y., Kim Y.S., Yi J.-S., Bae Y.-S. (2009). Platycodin D and 2 ″-o-acetyl-polygalacin D2 isolated from Platycodon grandiflorum protect ischemia/reperfusion injury in the gerbil hippocampus. Brain Res..

[121] Lee M.R., Yun B.S., In O.H., Sung C.K. (2011). Comparative study of Korean white, red, and black ginseng extract on cholinesterase inhibitory activity and cholinergic function. J. Ginseng Res..

[122] Kim Y., Kim S., Markelonis G., Oh T. (1998). Ginsenosides Rb1 and Rg3 protect cultured rat cortical cells from glutamate-induced neurodegeneration. J Neurosci Res 53: 426-432. J. Neurosci. Res..

[123] Wu J., Jeong H.K., Bulin S.E., Kwon S.W., Park J.H., Bezprozvanny I. (2009). Ginsenosides protect striatal neurons in a cellular model of Huntington’s disease. J. Neurosci. Res..

[124] Xu C.-L., Wang Q.-Z., Sun L.-M., Li X.-M., Deng J.-M., Li L.-F., Zhang J., Xu R., Ma S.-P. (2012). Asiaticoside: Attenuation of neurotoxicity induced by MPTP in a rat model of Parkinsonism via maintaining redox balance and up-regulating the ratio of Bcl-2/Bax. Pharmacol. Biochem. Behav..

[125] Chen X.-C., Zhu Y.-G., Zhu L.-A., Huang C., Chen Y., Chen L.-M., Fang F., Zhou Y.-C., Zhao C.-H. (2003). Ginsenoside Rg1 attenuates dopamine-induced apoptosis in PC12 cells by suppressing oxidative stress. Eur. J. Pharmacol..

[126] López M.V.N., Cuadrado M.P.G.-S., Ruiz-Poveda O.M.P., Del Fresno A.M.V., Accame M.E.C. (2007). Neuroprotective effect of individual ginsenosides on astrocytes primary culture. Biochim. Biophys. Acta (BBA)—Gen. Subj..

[127] Qian Y.-H., Han H., Hu X.-D., Shi L.-L. (2009). Protective effect of ginsenoside Rb1 on β-amyloid protein (1-42)-induced neurotoxicity in cortical neurons. Neurol. Res..

[128] Shin E.-J., Koh Y.H., Kim A.-Y., Nah S.-Y., Jeong J.H., Chae J.-S., Kim S.C., Yen T.P.H., Yoon H.-J., Kim W.-K. (2009). Ginsenosides attenuate kainic acid-induced synaptosomal oxidative stress via stimulation of adenosine A2A receptors in rat hippocampus. Behav. Brain Res..

[129] Shin E.-J., Nabeshima T., Suh H.-W., Jhoo W.-K., Oh K.-W., Lim Y.-K., Kim D.S., Choi K.H., Kim H.-C. (2005). Ginsenosides attenuate methamphetamine-induced behavioral side effects in mice via activation of adenosine A2A receptors: Possible involvements of the striatal reduction in AP-1 DNA binding activity and proenkephalin gene expression. Behav. Brain Res..

[130] Li L., Liu J., Yan X., Qin K., Shi M., Lin T., Zhu Y., Kang T., Zhao G. (2011). Protective effects of ginsenoside Rd against okadaic acid-induced neurotoxicity in vivo and in vitro. J. Ethnopharmacol..

